# Exploring Manipulated Prescribed Medicines for Novel Leads in 3D Printed Personalized Dosage Forms

**DOI:** 10.3390/pharmaceutics17020271

**Published:** 2025-02-18

**Authors:** Wouter Pannekoek, Eveline E. M. van Kampen, Frank van Tienen, P. Hugo M. van der Kuy, Elisabeth J. Ruijgrok

**Affiliations:** 1 Erasmus MC, Department of Hospital Pharmacy, University Medical Center, 3015 GD Rotterdam, The Netherlandsf.vantienen@erasmusmc.nl (F.v.T.); e.j.ruijgrok@erasmusmc.nl (E.J.R.); 2 Apotheek HaGi, 3371 AR Hardinxveld-Giessendam, The Netherlands; 3 Erasmus MC Sophia Children’s Hospital, Department of Hospital Pharmacy, University Medical Center, 3015 GD Rotterdam, The Netherlands

**Keywords:** three-dimensional, printing, prescriptions, child, hospitals, pharmacies, pharmaceutical preparations, retrospective studies, Netherlands, psychotropic drugs, delivery of health care

## Abstract

**Background:** On-demand personalized drug production is currently not addressed with large-scale drug manufacturing. In our study, we focused primarily on identifying possible active pharmaceutical ingredients (APIs) for 3D Printing (3DP) in the current healthcare setting. **Methods**: We conducted a retrospective cross-sectional study in the Netherlands using three different sources; community pharmacies (n = 5), elderly care homes (n = 3), and the Erasmus MC Sophia Children’s Hospital. The primary endpoint was the percentage of prescriptions of medication manipulated before administration, thereby being a candidate for 3DP. Around a million prescriptions were analyzed in our study. **Results**: This study shows that around 3.0% of the prescribed drugs dispensed by Dutch community pharmacies were manipulated before administration, while around 10.5% of the prescribed drugs in the Erasmus MC Sophia Children’s Hospital were manipulated prior to administration. **Conclusions**: With our study, we show that the most manipulated drugs come from the groups of constipation, psychopharmaceutical, cardiovascular, and anti-infectant drugs. Successful introduction of a compounded API drug by 3DP does not only rely on the API, but it also comes with an optimal balance between technical, economic as well as societal impact factors. Our study gives direction for potential future research on the introduction of 3DP of medicine in the healthcare setting.

## 1. Introduction

Currently, large-scale drug manufacturing by traditional methods lacks the ability of on-demand personalized drug production. Magistral compounded medicine by additive manufacturing or so-called 3D-pharmaprinting (3DP) can make a significant contribution towards addressing the demand for personalized medicine [1]. In the last decade, there has been a significant growth in scientific publications on 3DP of personalized medicine [2]. However, the implementation of compounded medicines by 3DP into daily healthcare remains a challenge, as illustrated by the use case published by X. Rodríguez-Maciñeiras et.al. [3]. A set of critical factors, such as regulatory, economic, ethical, and organizational factors, need to be overcome to use magistral compounded medicines by 3DP in healthcare organizations as described by Beer et al. [4]. In this study, we focus on detecting promising active pharmaceutical ingredient (API) for compounding medicine by 3DP. 

Defining a viable opportunity for 3DP of medicine is not merely an economic challenge. As with most potential innovative technologies, a successful introduction is a technical-economic issue requiring a techno-economic assessment [5]. The potential scale of the market, as well as the technological readiness level (TRL) of the technology, determine the success of the introduction of a new technique. 3DP of medicine could entail multiple different additive manufacturing techniques [1,6]. Several off-the-shelf printers for 3DP of medicine in the pharmacy are currently available, such as; the M3dimaker of FabRX [7], MED of Triastek [8], ZipDose of Aprecia [9], Flexdose of DiHeSys [10] and the DoseRx1 of Dosermedical [11]. The current TRL of the technique for 3DP in the pharmacy is therefore between TRL 7 and TRL 8, meaning they demonstrated added value, but did not reach the optimal deployment phase. To bring 3DP to a higher TRL level, our focus is on the identification of potential medicines for 3DP in the healthcare sector.

Suitable candidates for compounded medicine by 3DP would be medicinal dosage forms that are manipulated. In other words, APIs require the handling of a patient or healthcare professional before administration for specific patient groups and thereby introduce a risk of inadequate dosing. The 3DP-technology selection based on the different techniques available could better be performed when the API is already identified. We used the frequency of manipulations of different APIs in the healthcare setting as a surrogate for a potential economically viable scale. These APIs are currently not available in a registered product that meets the specific needs of the individual patient, while there is a large demand for adjusted dosages in the healthcare setting.

Currently, multiple patient groups are not adequately treated with the registered products of the pharmaceutical industry. Examples of these groups are pediatric patients [12,13,14,15,16,17,18] and frail elderly [19,20]. Children have a rapidly changing body [16,17], while frail elderly are dealing with multiple comorbidities as well as a changing body compared to an adult [19,20]. Therefore, both children and frail elderly need a different dosage strength than the commercially available medicines and would therefore benefit from personalized medicine. Lastly, long-term possibilities of 3DP could be personalized medicine based on patient factors, such as genetics, patient parameters, lifestyle, and other factors [1,21,22].

Therefore, we hypothesize that suitable candidates for compounded medicine by 3DP, are APIs that are currently being manipulated for children and frail elderly to fit their specific needs. To identify manipulated APIs for the larger total population we extracted data from the community pharmacies. For the identification of the APIs for pediatric patients and the frail elderly, we will use the extracted data from the Erasmus MC Sophia Children’s Hospital and nursing homes. 

## 2. Materials and Methods

### 2.1. Data Collection and Study Parameters

The primary objective of this study is to identify manipulated prescribed medicines that are candidates for 3DP dosage forms. Therefore, a retrospective cross-sectional study was conducted in the Netherlands. This study was approved as non-WMO by the ethical committee of Erasmus MC Rotterdam (MEC-2022-0169). This study was conducted according to the principles of the Declaration of Helsinki (version 2013) and following the General Data Protection Regulation (GDPR or Dutch AVG).

From different electronic healthcare information systems, all prescription data from 2019 (1st of January until 31st of December) were anonymized and collected. No prescription data were excluded. We used a dataset from 2019, as prescription data from this year was not influenced by the COVID-19 pandemic. The data came from three different sources consisting of community pharmacies (n = 5), elderly care homes (n = 3), and the Erasmus MC Sophia Children’s Hospital. Community pharmacies were selected with a geographical spread over the Netherlands. Due to limitations of the system used by the elderly care homes, only prescriptions active on the day of extraction could be retrieved. Sites were selected to cover as many diverse patient groups as possible: the general population of all ages, the elderly (older than 65 years), and children. The study population from Erasmus MC Sophia’s Children’s Hospital consists of children (ages between 0 and 18 years) admitted to the medium care unit (MCU), neonatal intensive care (NICU), and pediatric intensive care unit (PICU) of Erasmus MC Sophia Children’s Hospital in Rotterdam.

Collected data included information regarding the patient (sex, age, patient identification number), drug characteristics (name of API, name of a commercial product, Anatomical Therapeutic Chemical (ATC) code) [23], and drug administration records (dose, route of administration, free text for the prescriber). In addition, source-specific information was collected, for example, the length of hospital admission at Erasmus MC Sophia Children’s Hospital.

### 2.2. Data Cleaning

For the scope of this study, we define manipulated medicines as follows: Prescribed drugs that require the handling of a patient or healthcare professional before administration, which introduces a risk of inadequate dosing. Medication is therefore considered manipulated when: the prescribed dose is not equal to the commercially available dose, the patient suffers from swallowing issues, or when the patient is intubated, see Appendix A. These manipulations can range from simple handling, such as withdrawing a specific volume of liquid from a larger bottle to more complex handling such as crushing a tablet, dissolving the powder in a syringe, and keeping the mixture in suspension during administration. 

The included community pharmacies use Pharmacom (PharmaPartners B.V.) as their information system. They provided their complete dispensing data, which includes more information than only prescription data. Therefore, communication notes such as purchasing medication, calling for advice, and an invitation for a yearly evaluation were filtered out. Data from the elderly care homes were extracted from Medimo (Medimo B.V.), their electronic prescribing system. From this registration system, we could filter in advance for manipulated tablets, which included either half or quarter tablets administered to patients. No further filtering steps were needed. The limitation of this system was that we could only retrieve the prescription data of the day of extraction and only tablets. Data from Erasmus MC Sophia’s Children’s Hospital was extracted from their electronic hospital information system, HiX 6.2 (ChipSoft B.V.). All medication orders were provided, including gestational age at birth, duration of hospitalization, and usage of a feeding tube. The sparingly featured non-medication entries were filtered out, for example, instructions to change bandages. 

Similar filter strategies were used for the datasets of the community pharmacies and the Erasmus MC Sophia Children’s Hospital. Analysis was conducted using R (R Core Team, 2020), RStudio (Rstudio Team, 2020), and the tidy verse package of Wickham, 2017 (Free Software Foundation, Inc., Boston, MA, USA). First, the data sets were trimmed by removing columns featuring information not used during our analysis, for example, the name of the prescriber or the ward was removed. Secondly, a set of filters was applied in a predefined order which is listed in Appendix B. Text fields were scanned for keywords based on the inclusion criteria, in order to prevent false negative results. The text fields specified either if the medication was taken via an oral, rectal, or vaginal route (when filtered on administration routes) or if the product was a capsule or tablet (when filtered on products unsuitable for printing). After these filtering steps, only prescriptions requiring manipulation before administration remained. Those prescriptions were sorted based on their dosage form by ATC code. 

### 2.3. Data Analysis and Endpoints

After data cleaning, the prescriptions were analyzed per source, in bulk, and per dosage form. The primary endpoint of this study is the percentage of prescriptions of medication manipulated before administration, thereby being a candidate for 3DP. This was measured as a percentage of instances in which the dose prescribed was either unequal to the commercially available dose or had to be manipulated before administration. A list detailing the prevalence of individual drugs was constructed.

In addition, differences in manipulation rates between sexes were investigated, ATC codes were used to track different groups of medication and a descriptive evaluation of prescriptions of medication manipulated before administration was conducted per age group. Age groups were defined as follows:

- Children:
- Neonates, both preterm and term: from the day of birth up to 27 days;
- Infants (or toddlers): from 1 month (28 days) to 23 months;
- Children: from 2 years to 11 years; and
- Adolescents: from 12 years to less than 18 years.
- Adults: 18 until 65 years of age
- Elderly: older than 65 years of age.

## 3. Results

### 3.1. Data Cleaning

**Figure A1:**
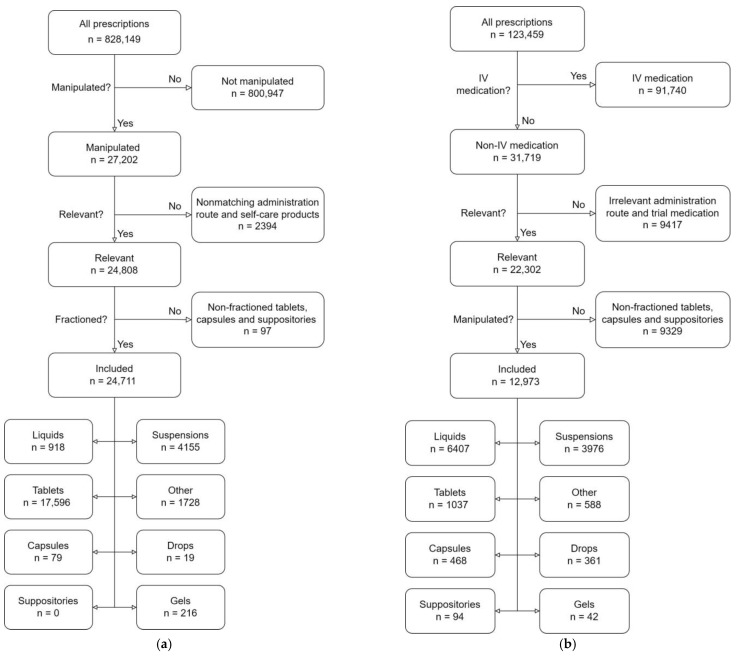
(**a**) Flowchart inclusions in the community pharmacies. (**b**) Flowchart inclusions in the Erasmus MC Sophia Children's Hospital.

The data sets of the three different sources were filtered individually, due to their varying data structures. This required a tailored approach per source, with the order of steps not being consistent. The guiding principle of following the same steps per dataset was the base for filtering the data as shown in Figure A1a, Appendix B. Across all community pharmacies, 3.0% of prescriptions were manipulated. From the elderly homes we used only prescriptions of manipulated oral dosage forms, and therefore no percentage can be calculated. In the Erasmus MC Sophia Children’s Hospital, 10.5% of all administrations were manipulated.

The data collection from five community pharmacies yielded 828,149 prescription orders, see Figure A1a, Appendix B. Most of the prescriptions (800,947) were administered as prescribed and filtered out. Of the 27,202 entries that remained, another 2394 were excluded after free text mining. The filtered community pharmacy dataset contained 24,711 entries, which corresponds to 3.0% of the starting data set.

The data of the elderly care homes was prefiltered on non-integer amounts of tablets, so it was cleaned beforehand. This results in a reduced number of prescriptions in the starting data set and the lack of manipulation rates calculated.

The data extraction from the Erasmus MC Sophia Children’s Hospital yielded 123,459 prescription orders, see Figure A1b, Appendix B. Hereof, 91,740 were filtered out based on non-IV medication, after which they were filtered on unsuitable administration route for 3DP (e.g., creams) or ATC code. More than half of the remaining 31,719 entries (18,746) did not contain any information related to splitting, partial administration, or any other kind of manipulation. Thereby, the final Erasmus MC Sophia Children’s Hospital data set contained 12,973 entries, which is 10.5% of all prescription orders. If we look in more detail and only account for the oral, vaginal, and rectal administration routes, 40.9% of those prescriptions were manipulated before administration.

### 3.2. Data Analysis and Endpoints

#### Dosage Forms

Of the 24,711 manipulated prescriptions in community pharmacies, 17,596 (71.2%) were tablets, see Figure A1a, Appendix B. Three other large groups were oral suspensions (4155 = 16.8%), miscellaneous or other (2042 = 8.3%), and oral solutions (918 = 3.7%). “Miscellaneous or other” was used for cases with diverging names or incorrect labeling. In practice, this miscellaneous group mostly consisted of oral liquids and suspensions.

When we look at the pediatric population under 18 years of age, we yielded 2182 manipulated prescriptions, from the total of 25,609 pediatric prescriptions (8.5%) in community pharmacies. This subset has a different distribution compared to the total population in community pharmacies: oral suspensions made up the majority (1221 = 56.0%), followed by miscellaneous/other (371 = 17.0%), tablets (352 = 16.1%) and oral solutions (195 = 8.9%).

The elderly population of community pharmacies is responsible for the majority of prescriptions (443,684 prescriptions). Of these, 13,451 were manipulated, also representing 3.0% of the total prescriptions in this group.

In the Erasmus MC Sophia Children’s Hospital, the largest group was oral solutions: 6407 (49.4%) out of 12,973 manipulated prescriptions total followed by oral suspensions (3976 = 30.8%), tablets (1037 = 8.0%), and miscellaneous or other (1553 = 12.0%), see Figure A1b Appendix B.

**Figure 1:**
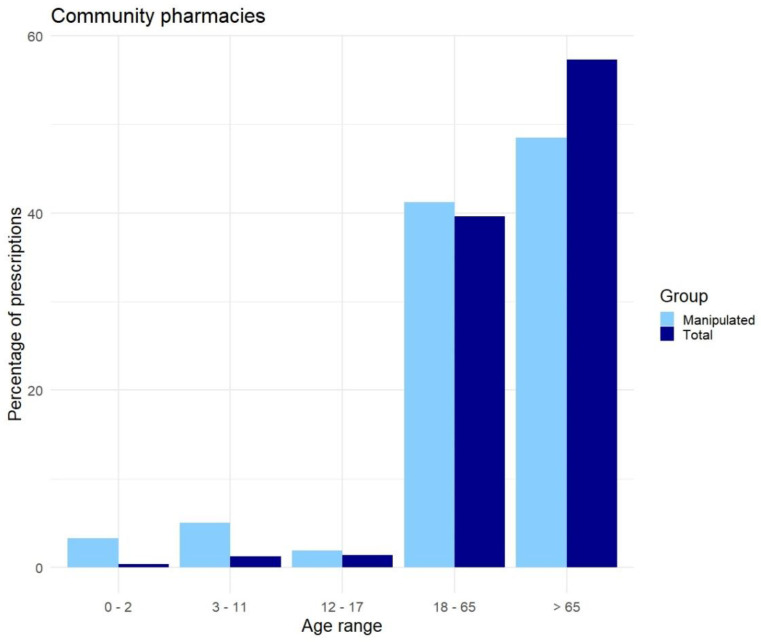
Overview of the source data (dark blue “Total”) and the manipulated prescriptions (light blue “Manipulated”) per age group, in community pharmacies.

As can be seen in Figure 1, elderly (>65 years) in the community pharmacy use most of the medication (57.3%), but not of the manipulated drugs (48.5%) based on our definition of manipulation. While the younger population (<18 years) uses less medication compared to the older population (3.1%); however, the younger population contributes to 10.2% of the manipulated drugs. 

In total 516 different manipulated APIs were identified in community pharmacies. Less than a quarter were used in the pediatric population of community pharmacies. As can be seen in Table 1, the most manipulated medication in community pharmacies was lactulose syrup, followed by oxazepam, dipiperone, haloperidol, prednisolone, lorazepam, mirtazapine, levothyroxine, amoxicillin, and valproic acid. For children in general amoxicillin was the most manipulated API, followed by desloratadine, methylphenidate, nystatin, amoxicillin/clavulanic acid, omeprazole, azithromycin, cotrimoxazole, nitrofurantoin, and ranitidine. For the elderly aged above 65, the most frequently manipulated APIs were lactulose, oxazepam, levothyroxine, prednisolone, and metoprolol.

**Figure 2:**
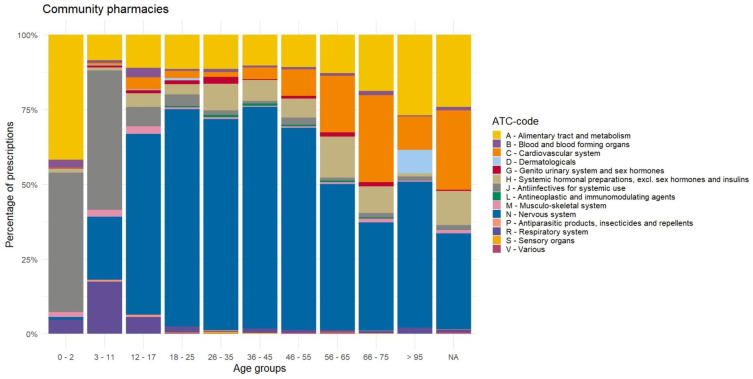
Distribution of manipulated prescriptions per ATC-code by age, for the community pharmacies.

**Figure A2:**
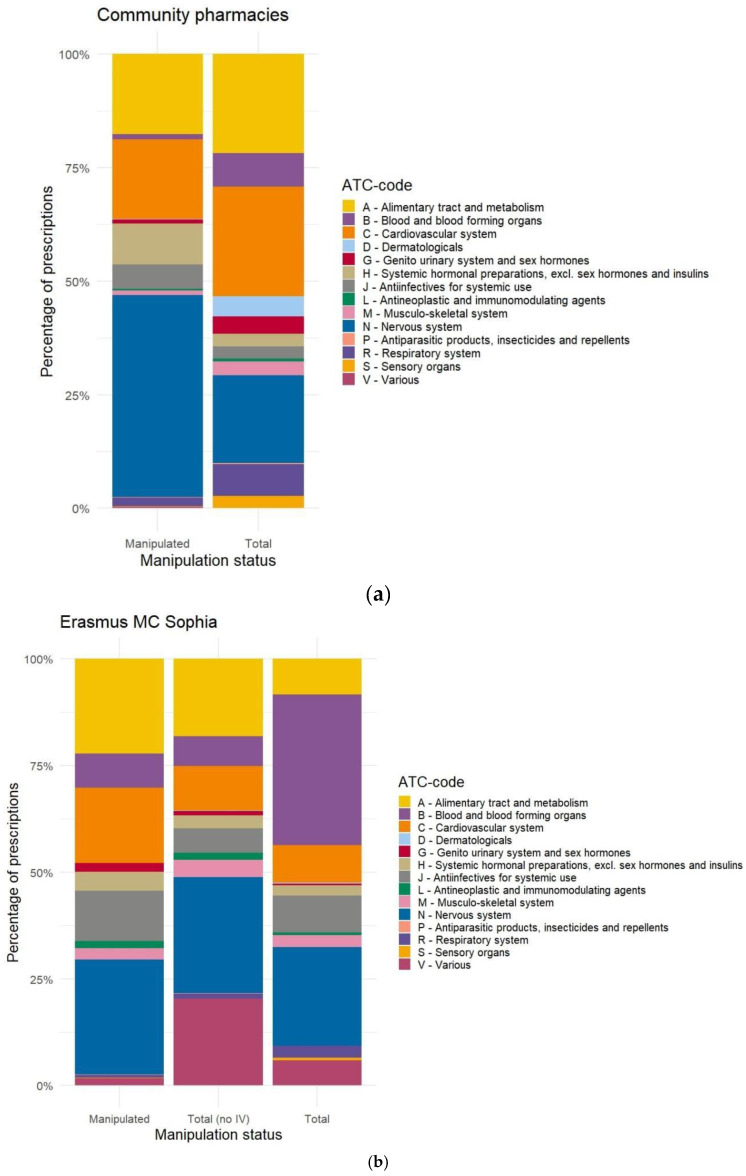
(**a**) Distribution of manipulated prescriptions and source data (total) based on ATC-code in the community pharmacies. (**b**) Distribution of manipulated prescriptions (manipulated), the total prescriptions without IV, and the source data (Total) based on ATC-code, in the Erasmus MC Sophia Children’s Hospital.

**Figure A3:**
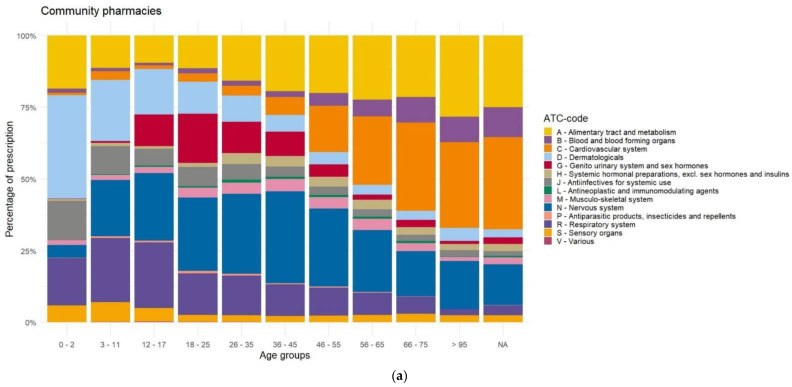

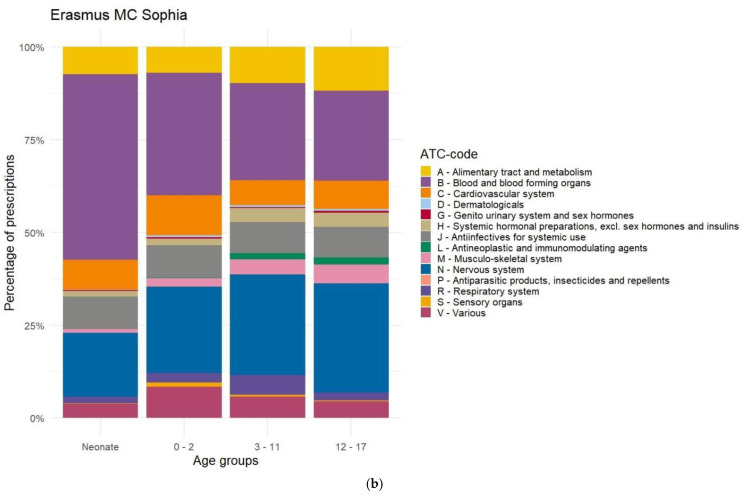

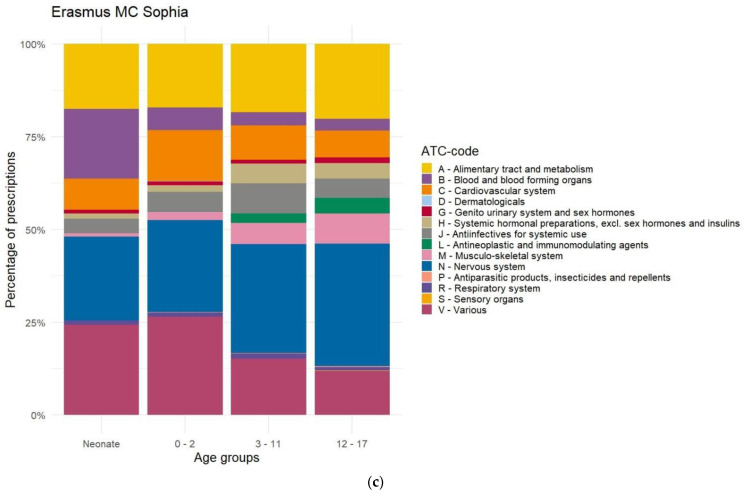
(**a**) Distribution of prescriptions based on ATC-code ranked by age group before filtering on manipulation, in the community pharmacies. (**b**) Distribution of prescriptions based on ATC-code ranked age group before filtering on manipulation, in the Erasmus MC Sophia Children’s Hospital. (**c**) Distribution of prescriptions without IV before filtering on manipulation based on ATC-code ranked by age group, In the Erasmus MC Sophia Children’s Hospital.

Analyzing the distribution of manipulated prescriptions (Figure 2 and Appendix C, Figure A2a) versus the total prescribed dosages in (Appendix C, Figure A2a and Appendix D, Figure A3a), define ATC group are defined that are more prone to manipulation in total and per age group. Overall, we see that most constipation, psychopharmaceutical, cardiovascular, and anti-infectant drugs are manipulated. 

When analyzing the prescriptions of manipulated medication of the community pharmacy per age group, we identified a preference for antibiotics and anti-infective drugs (ATC-class J) for younger people and drugs on the nervous system (ATC-class N) for adults, as can be seen in Figure 2. Prescriptions for the respiratory system (ATC-class R) are more frequently manipulated from the age group 3–11 years compared to the age group 0–2 years, while those drugs are almost never manipulated for adults. Furthermore, we see that in the age group 0–2 years there is a high amount of manipulation of drugs for the alimentary tract and metabolism (ATC-class A). Additionally, we identified a trend in elderly patients of increasing use of manipulated drugs on the cardiovascular system (ATC-class C), systemic hormones, excluding sex hormones and insulins (ATC-class H), and the alimentary tract and metabolism (ATC-class A). The number of manipulated drugs for the nervous system (ATC-class N) for the ages 18–65 (66.5%) stands out.

In the Erasmus MC Sophia Children’s Hospital, this list shows different types of medication (see Table 1). In total, 246 different APIs were identified as being manipulated. Whereof, macrogol was the most manipulated API, followed in order by caffeine, furosemide, esomeprazole, lorazepam, ferro fumarate, paracetamol or acetaminophen, cotrimoxazole, amoxicillin or clavulanic acid and hydrochlorothiazide. 

**Figure A4:**
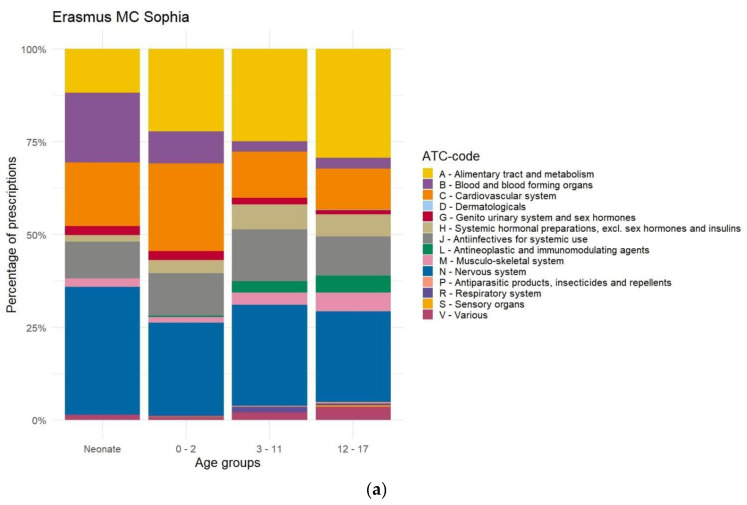

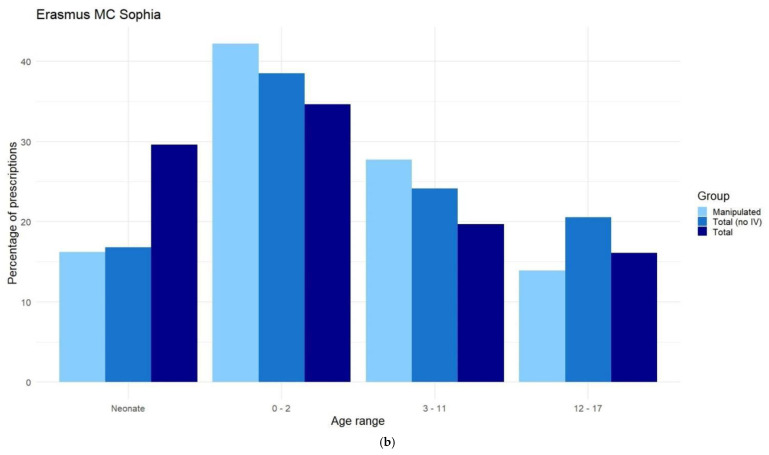
(**a**) Distribution of prescriptions based on ATC-code ranked by age group before filtering on manipulation, in the Erasmus MC Sophia Children's Hospital. (**b**) Distribution of manipulated prescriptions (light blue, manipulated), the prescriptions suitable for 3DP (blue, total (no IV)), and the source data (dark blue, Total) ranked by age in the Erasmus MC Sophia Children's Hospital. Total (adjusted) is filtered to only include relevant routes of administration, with no other filters applied.

For the Erasmus MC Sophia Children’s Hospital, the overview in relation to the ATC class is shown in Appendix C, Figure A2b. In this figure, we filtered out the IV route of administration, so we retain dosage forms suitable for 3DP. The differences in age groups for the total prescriptions can be found in Appendix D, Figure A3b. The total prescriptions per age group without IV can be found in Appendix D, Figure A3c and the manipulations per age group can be found in Appendix E, Figure A4a. In the hospital setting manipulated cardiovascular drugs, drugs for the alimentary tract system (ATC-class A) and drugs for the nervous system (ATC-class N) were more prevalent compared to the outpatient setting. Additionally, drugs for the blood and blood-forming organs (ATC-class B) were manipulated frequently, especially for neonates. In the community pharmacy antibiotics and anti-infectant drugs (ATC-class J) were more prevalent. In the Erasmus MC Sophia Children’s Hospital, we found a clear decrease in the manipulation of prescriptions when children were getting older (Appendix E, Figure A4b).

The distribution of different drugs within elderly care homes was also evaluated in Table 1. In total, 18 different APIs were identified as being manipulated. Oxazepam was the most manipulated API, followed in order by haloperidol, prednisolone, lorazepam, risperidone, macrogol, morphine, mirtazapine, metoprolol, and tetrabenazine.

**Figure A5:**
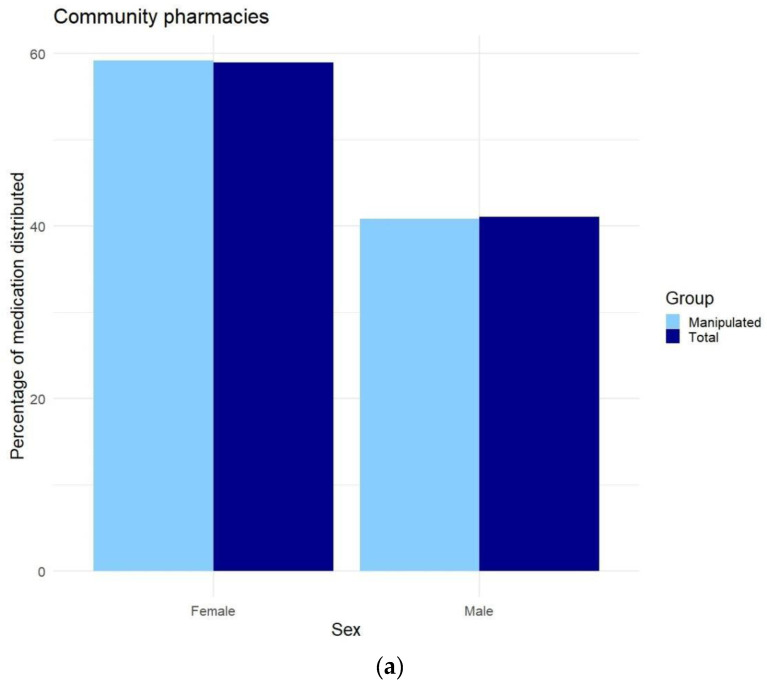

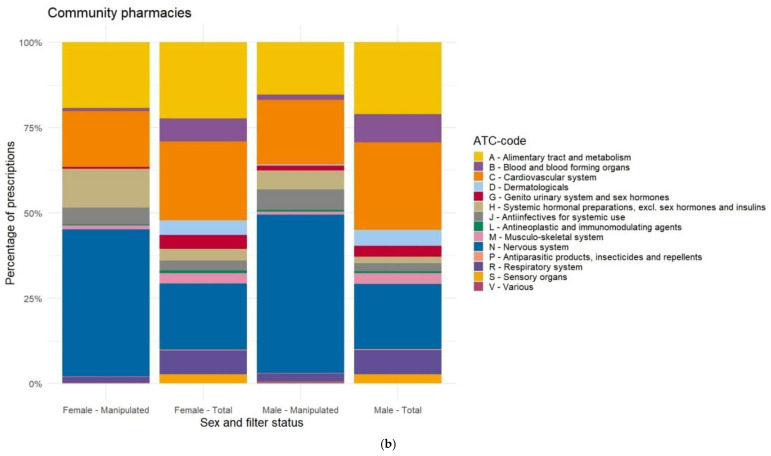
(**a**) Comparison of manipulated prescriptions versus total source data ranked by gender, in the community pharmacies. (**b**) Distribution of prescriptions based on ATC-code ranked by gender before (total) and after (manipulated) data cleaning, in the community pharmacies.

When we compare prescriptions based on sex, we see that 59.0% of all drugs were prescribed to women in the general population (Appendix F, Figure A5a). Additionally, manipulated drugs were more often prescribed to women. When we investigated in more detail, we saw that manipulating hormones (especially thyroid hormones) was more prevalent in women, while manipulating cardiovascular and psychopharmaceutic drugs was slightly more prevalent in men (Appendix F*,* Figure A5b). 

## 4. Discussion

This study shows that 3.0% of the prescribed drugs dispensed by community pharmacies in the Netherlands need to be manipulated before administration, and 10.5% of the prescribed drugs are in the Erasmus MC Sophia Children’s Hospital. If we only look at oral, vaginal, and rectal administrations, the percentage is 40.9%. The drugs that are most commonly being manipulated are constipation, psychopharmaceutical, cardiovascular, and anti-infectant drugs. 

The results of drug consumption in our research population are comparable with the population data available in the Netherlands on drug consumption (data Stichting Farmaceutische Kengetallen) [24]. Therefore, we can assume that our data analysis of manipulated drugs can be extrapolated to the Dutch population in general. Extrapolation of our results beyond the Netherlands to the European Union might be possible. However, we should take differences in the availability of registered drugs and dosages into consideration. Additionally, differences in prescribing behavior should be taken into account. For example, antibiotic prescriptions vary widely between European countries [25]. Nonetheless in this study, we found that APIs with a potential economically viable scale for compounding in the Netherlands by 3DP are psychopharmaceutical, cardiovascular, and anti-infectant drugs. This might also be the case in a European or broader context. 

Besides the economically viable scale, we should also consider the regulatory and ethical issues involved with the introduction of the technique of printing for compounded medicine by 3DP [4]. Within the medical–ethical discourse, using the “Precaution Principles” is common practice when introducing new techniques without a clear and established regulatory and ethical framework [26,27]. One should therefore consider the “societal benefits” associated with the problem in the healthcare sector they are trying to solve with the introduction of compounded medicine by 3DP. Societal acceptance by solving an important problem will help to address regulatory and ethical issues involved with the introduction of a new technique, especially when the risks associated are low and there are no other solutions available to solve the problem. The societal acceptance of 3DP might even increase when unmet medical needs with a high societal impact, which are not yet addressed by the pharmaceutical industry, are solved with the technique. Thereby, the best potential successful introduction of a compounded API drug by 3DP is an optimal balance between technical, economic as well as societal impact factors.

One limitation of this study was extracting data from different IT systems. When interpreting the results of this study one should take into account that we used datasets from different IT systems in the healthcare setting. The different systems have limited possibilities for the extraction of different data parameters. The most extensive search of different available data parameters was executed in the Erasmus MC Sophia Children’s Hospital. The possible data parameters that could be extracted from the community pharmacy were reduced compared to the pediatric hospital. The available data parameters in the nursing homes were even more restricted, as only a search of the quarter and half tablets administered to patients could be executed. Therefore, when analyzing the results, we were limited by the data fields from the different IT systems available to us. This restricted us from adequately identifying the administered drugs that were being manipulated due to the introduction of a certain margin of error based on the limitations of the available and extractable data. We analyzed the label-text of the community pharmacy and daily defined dose in the healthcare systems, therefore manipulation that occurred before administration that was not recorded in the system was not detected in this study. Additionally, the lack of the ICPC-code (International Classification of Primary Care-code) of the prescribed drugs hindered us from further analysis of the data in combination with the diagnosis and thereby identifying specific morbidities for the introduction of potential compounded medicine by 3DP. 

A second limitation is that this study was performed in the Netherlands. Therefore, it should be interpreted within the context of the Dutch healthcare setting and healthcare sector. An example of this is the high amount of drug shortages in the Netherlands as exemplified by the recorded data of the Royal Dutch Pharmacists Association (KNMP) [28]. Additionally, the preference policy of healthcare insurance in the Netherlands for generic brands should be considered. Based on the Dutch multidisciplinary guideline for substitution of medicine [29] and the ‘*Handleiding Geneesmiddelensubstitutie*’ (Dutch pharmacist manual for substitution of medicine) [30], the prescription with a medical need for a brand is also accepted in the Netherlands. The high number of manipulations of ivabradine and levothyroxine found were a result of prescribing branded drugs due to medical urgency, while generic alternatives were available. 

The third limitation is that our methodology has a blind spot for potential APIs for 3DP which currently are not available or suitable at all. Hereby we allude to potential APIs which currently cannot be dosed adequately by manipulating existing registered drugs and APIs that have no established framework of personalized medicine in the current clinical setting.

Defining the manipulation of drugs poses a difficulty as it has no established criteria, therefore this term is defined differently in each study [31]. One could argue that manipulation is an off-label use, or non-conformity to the SMPC (Summary of Product Characteristics)-text, of the different registered drugs. However, this would exclude the labor-intensive and potentially inadequate dosing of drugs. For example, halving tablets could be in line with the SMPC text but is labor-intensive and has a risk of inadequate dosing [13]. This also applies to the administration of suspensions and other liquids [32] and the crushing of tablets before administration. Therefore, we chose to define manipulated drugs as follows: Prescribed drugs that require the handling of a patient or healthcare professional before administration, which introduces a risk of inadequate dosing. Furthermore, we deem that drugs dosed via oral, vaginal, and rectal administration routes can be replaced by compounded drugs with 3DP techniques and therefore excluded other administration routes such as IV administration. Therefore, our study used a definition of manipulation of drugs based on our research question to identify potential APIs for 3DP. 

Based on our definition of manipulation we found that in the general population, the elderly (>65 years) use most of the medication (57.3%) but use relatively less manipulated drugs (48.5%). The younger population (<18 years) uses little medication compared to the elderly (3.1%); however, the younger population contributes to 10.2% of the prescriptions of manipulated drugs. These results were unexpected, as a larger number of manipulations among the elderly was expected due to their changing body and a higher number of comorbidities among the elderly. [19,20]

The trends in shifting ATC classes that are being manipulated could be partially explained by using antibiotics as liquids and suspensions for children and the increase in cardiovascular diseases and changing bodies and metabolism in the elderly. The increase in respiratory drugs during childhood can be explained by anti-histaminic drug usage according to the Kinderformularium (Dutch Pediatric Formulary [33]). The increase of manipulated drugs in the alimentary tract and metabolism could mostly be explained by an increase of use medicine to treat constipation for both young children and the elderly. The number of manipulated drugs for the nervous system for the ages 18–65 (66.5%) stands out clearly. 

When analyzing the class of manipulated drugs for the nervous system we can identify benzodiazepines (oxazepam, lorazepam, diazepam), anti-psychotics (pipamperone, haloperidol, risperidone), anti-depressants (mirtazapine, sertraline, paroxetine), anti-epileptics (carbamazepine, valproate) and drugs for ADHD (methylphenidate) as the main drugs that are being manipulated from this class. What this group and these drugs have in common is a subjective treatment goal and a difficult balance between treatment results and side effects. For anti-depressants in adult and older patients, it has been shown by Cipriani et al. and Krause et al. that the balance between acceptance and efficacy is an important factor in determining the choice of drug therapy [34,35]. Additionally, these results can be partially explained by the off-label use and/or reduced dosing of psychopharmaceuticals in the Netherlands to treat, among others, sleeping disorders (benzodiazepines, quetiapine, amitriptyline, and mirtazapine) [36,37]. Lastly, some psychopharmaceuticals can induce dependency in patients and result in withdrawal symptoms, resulting in a therapeutic rationale to use manipulated medicine in order to phase out medication [38,39,40,41,42]. The shift of manipulation of drugs towards mostly benzodiazepines, anti-psychotics, anti-depressants, and anti-epileptics manifests itself again and is even more pronounced by the frail elderly in nursing homes. On the other hand, the general aging population showed a decrease in psychopharmaceutical drugs and an increase in cardiovascular drugs and drugs to treat constipation. This reversing trend among frail elderly can be explained by the clinical relevance and necessity of deprescribing drugs due to their vulnerability to harm from medication [43]. The clinical relevance of potential harm among the elderly could lead to the future development of personalized 3DP drugs for frail elderly.

The dataset Erasmus MC Sophia Children’s Hospital is characterized by a significant amount of parenteral medication administration. These results are in line with the findings of Heneghan et al., who also found a high amount of prescription of intravenous nutrition products (ATC-class B) in pediatric ICUs [44]. This is due to the fact that those children are primarily hospitalized in high- or medium-intensity care units. From the analysis of age distribution, we found a clear decrease in manipulated prescriptions for older children at the Erasmus MC Sophia Children’s Hospital. This trend is also described in the study of Balan et al. [45]. When comparing the results from the general younger population in the community pharmacy to the results from the Erasmus MC Sophia Children’s Hospital, we found a clear difference in manipulated API. This is not surprising as Erasmus MC Sophia Children’s Hospital is a tertiary pediatric clinic. Furthermore, most of the children in the NICU are intubated for which manipulations of drugs are necessary. The cardiovascular drugs, drugs for the alimentary tract system, and drugs for the nervous system were more frequently manipulated than the antibiotics and anti-infectant drugs in the hospital setting compared to the outpatient setting. Additionally, drugs for the blood and blood-forming organs were manipulated frequently, especially for neonates. Neonates are still the most neglected patient group when it comes to the prevalence of registered drugs [46]. Heneghan et al. described that the most prevalent indications in the ICU are Respiratory disorders, injury/poisoning, endocrine/nutritional/immunological disorders, nervous system disorders, and circulatory disorders [44], which illustrates the need for different drugs when compared to the community pharmacy setting. 

Our analysis comparing prescriptions for men and women showed that 59.0% of the drugs were prescribed to women in the community pharmacy. This is in line with studies conducted in Germany [47], Italy [48], Spain [49], and the USA [50]. The same distribution between genders was found for manipulated drugs. These differences in consumption of medicine based on gender can be explained by the higher number of diagnosed morbidities among women in the Netherlands [51,52], differences in diagnosis between men and women [53], and their lower perception of their health [54]. There were only minor differences in the manipulated APIs detected. Manipulations of hormones (especially thyroid hormones) were more prevalent in women, while manipulation of cardiovascular and psychopharmaceutical drugs was slightly more prevalent in men. These findings were unexpected based on the general discussion on women’s health and especially the recent findings of Vogel et al. that the current cardiovascular treatment should be reconsidered for women [55]. Considering there is a difference between men and women in prescribed medicine [47,48,49,50], it might be possible that a deeper statistical study of differences in the manipulation of drugs based on gender will identify a different need for personalized medicine. However, no large differences in the manipulation of drugs based on gender could be identified with our methodology in this study. This inconclusive result could either be due to a lack of awareness by prescribers about the historical focus of pharmaceutic research in the clinical development phase on male participants, or this could be due to a limited clinical relevance to dose drugs differently in men and women. Therefore, further research on the clinical relevance of personalized medicine based on gender is required. 

This study primarily focused on the potential API for compounding medicine by 3DP, to stimulate the development of the technique to a higher TRL level. Without the development of 3DP, we expect an increase in the manipulation of medication in the coming decade. We expect that there will be an increased demand for personalized medicine for psychopharmaceutical and cardiovascular drugs due to the aging of the population, whilst our results show an increase in manipulated cardiovascular and psychopharmaceutical drugs when people age. Lastly, we expect the scientific community to further identify personalized needs for different APIs and morbidities, which also will increase the demand for personalized medicine in the healthcare setting. Our study lays the ground for potential future research in different directions to address these developments and challenges. One direction for future research could be the identification of a lack of adequate pharmacotherapeutic therapies within specific patient populations and/or for specific morbidities. This could identify potential leads for new personalized therapies by 3DP of medicine. Another direction for future research could be determining the potential market scale and economic feasibility for compounding medicine of the identified APIs in combination with the different available 3DP techniques. Furthermore, our study does not adequately answer the question of what scale products are not being used as specified in the SMPC in the healthcare sector. Future research within this field would be the determination of the scale of off-label use of registered products within the healthcare setting. In summary, further research is required on potential personalized API for specific patient groups, the techno-economic feasibility of compounding medicine by 3DP, and determining the scale of off-label drug consumption.

## 5. Conclusions

With our study, we show that a substantial number of drugs need to be manipulated before use by patients. This number is higher in a tertiary clinic than in the community pharmacy population. Most manipulated drugs come from the group of constipation, psychopharmaceutical, cardiovascular, and anti-infectant drugs. We have listed suitable API candidates for the implementation of 3DP in the healthcare sector. Successful introduction of a compounded API drug by 3DP does not only rely on the API, but it also comes with an optimal balance between technical, economic as well as societal impact factors.

## Figures and Tables

**Table 1:** Overview of the top 25 most manipulated APIs (with ATC code and exact amount of prescriptions) for each (sub)set of the data.

	Community Pharmacy (All Ages)			Community Pharmacy (Elderly)			Community Pharmacy (Pediatrics)			Elderly Care Home			Sophia Children’s Hospital		
**1**	Lactulose	A	1933	Lactulose	A	1644	Amoxicillin	J	558	Oxazepam	N	30	Macrogol	A	1055
**2**	Oxazepam	N	1286	Oxazepam	N	818	Desloratadine	R	183	Haloperidol	N	26	Caffeine	N	961
**3**	Pipamperone	N	1254	Levothyroxine	H	811	Methyl-phenidate	N	144	Prednisolone	H	24	Furosemide	C	779
**4**	Levothyroxine	H	991	Prednisolone	H	519	Nystatin	A	121	Lorazepam	N	19	Esomeprazole	A	650
**5**	Haloperidol	N	716	Metoprolol tartrate	C	386	Amoxicillin/ Clavulanic acid	J	110	Risperidone	N	12	Lorazepam	N	575
**6**	Prednisolone	H	667	Lorazepam	N	380	Omeprazole	A	109	Levothyroxine	H	11	Ferrous fumarate	B	480
**7**	Lorazepam	N	662	Enalapril	C	379	Azithromycin	J	65	Macrogol	A	10	Paracetamol	N	452
**8**	Mirtazapine	N	648	Metoprolol succinate	C	365	Lactulose	A	64	Morphine	N	9	Cotrimoxazole	J	439
**9**	Amoxicillin	J	555	Bumetanide	C	341	Cotrimoxazole	J	62	Mirtazapine	N	9	Amoxicillin/ Clavulanic acid	J	369
**10**	Valproic acid	N	493	Haloperidol	N	328	Nitrofurantoin	J	62	Metoprolol tartrate	C	9	Hydrochlorothiazide	C	354
**11**	Metoprolol tartrate	C	470	Mirtazapine	N	305	Risperidone	N	59	Tetrabenazine	N	8	Phytonadione	B	320
**12**	Sertraline	N	452	Amlodipine	C	248	Ranitidine	A	54	Valproic acid	N	7	Spironolactone	C	295
**13**	Enalapril	C	406	Clonazepam	N	225	Clarithromycin	J	41	Trazodone	N	5	Prednisolone	H	276
**14**	Bumetanide	C	358	Ivabradine	C	200	Cetirizine	R	33	Spironolactone	C	5	Ibuprofen	M	230
**15**	Metoprolol succinate	C	335	Zolpidem	N	158	Ferrous fumarate	B	32	Baclofen	M	5	Propranolol	C	202
**16**	Nystatin	A	316	Diazepam	N	155	Ibuprofen	M	30	Carbidopa/ Levodopa	N	5	Tramadol	N	197
**17**	Carbamazepine	N	306	Sertraline	N	131	Macrogol	A	28	Metoprolol succinate	C	4	Levetiracetam	N	196
**18**	Methylphenidate	N	280	Nystatin	A	129	Aripiprazole	N	27	Furosemide	C	4	Oxybutynin	G	187
**19**	Amlodipine	C	262	Temazepam	N	128	Levetiracetam	N	24				Clonidine	C	184
**20**	Risperidone	N	260	Risperidone	N	123	Ondansetron	A	23				Amphotericin B	J	177
**21**	Diazepam	N	254	Spironolactone	C	116	Miconazole	A	17				Dexamethasone	H	175
**22**	Omeprazole	A	237	Cholecalciferol	A	112	Flucloxacillin	J	17				Nystatin	A	153
**23**	Zolpidem	N	237	Lisinopril	C	111	Levocetirizine	R	17				Azithromycin	J	148
**24**	Ivabradine	C	233	Sotalol	C	110	Pheneticillin	J	14				ORS	A	148
**25**	Paroxetine	N	221	Omeprazole	A	109	Dexamethasone	H	14				Midazolam	N	138

## Data Availability

The data presented in this study are available on request from the corresponding author. The raw data supporting the conclusions of this article will be made available by the authors on request. Restrictions apply to the availability of these data. Data were obtained from the Sophia Children’s Hospital, Apotheek Bergschenhoek, Apotheek HaGi, Apotheek Goverwelle, Apotheek De Meeuw, and Apotheek Sleeuwijk and are available from the authors with the permission of the third party.

## Appendix A. List of Manipulations per Drug Dosage Form

**Table A1:** List of manipulations per drug dosage form, table adjusted from Richey et al. [12].

Drug Dosage Form	Manipulation for Dose Accuracy
**Tablet**	Split/broken/cut and a segment given Crushed and a proportion of the powder given Dispersed in liquid and a portion of the liquid given
**Capsule**	Opened, dispersed in liquid, and a proportion of the liquid given Opened and a portion of the powder given
**Sachet (powder)**	Opened, dispersed in liquid, and a portion of the liquid given Opened and a proportion of the powder given
**Oral liquid**	Diluted and a proportion given (to make the measurement of a small dose volume easier)
**Suppository**	Cut/split and a segment given
**Intravenous injection**	Reconstituted or ready-prepared solution, further diluted to allow a smaller dose to be measured. Volume of fluid removed from IV container and given orally.

## Appendix B. Data Cleaning

*Filter Strategy for the Data of Erasmus MC Sophia Children’s Hospital:*

1. Administration routes that are suitable for printed medication included the following: oral, oral/inhalation, oromucosal, rectal, sublingual, and vaginal administrations.
2. Anatomical Chemical Therapeutic (ATC) codes (as maintained by the World Health Organization) that are not suitable for printed medication were removed; A01A, A06AG, D., G01, J06, J07, M02, P03, R01A, R03A, R03B, S01, S02, S03, V07, V08, V09, V10, V20, missing ATC value.
3. Partial administration, every entry that did not contain any indication of partial administration of a standard unit was removed. These indications include measures of weight, volume, fractions, named fractions, and numerical values.
4. To prevent accidental removal based on free text, all entries related to tubes, syringes, and dissolving tablets/capsules in water were stored in a separate dataset.
5. Based on the free text, entries were removed, primarily based on free text in the prescription instructions. This included a misspelling of previous removal criteria, anything related to test patients, anything other than prescription medication (bandages, non-prescription medication, sunscreen), internal notes (“discussed with the doctor”, “automatically repeat every three months”, “medication not dispensed”, etc.), medication used in clinical trials.
6. Afterward, the dataset containing entries related to tubes, syringes, and dissolving was added to the included dataset. Integer numbers were removed, such as “X pieces” in a text note.
7. The dose per unit and the dose per gift were calculated. If this did result in an integer number of units required, the entry was removed.

*Filter Strategy for the Data of Community Pharmacies:*

For community pharmacies, step 7 is performed before step 3, due to the limitation of the source data. In addition, steps 1, 4, and 6 were omitted.

*Filter Strategy for the Data of elderly homes:*

Filtering was not needed for the source data from elderly homes, as it was retrieved and filtered on manipulation.

## References

[1] Lind J., Kalvemark Sporrong S., Kaae S., Rantanen J., Genina N. (2017). Social aspects in additive manufacturing of pharmaceutical products. Expert Opin. Drug Deliv..

[2] Xue A., Li W., Tian W., Zheng M., Shen L., Hong Y. (2023). A Bibliometric Analysis of 3D Printing in Personalized Medicine Research from 2012 to 2022. Pharmaceuticals.

[3] Rodríguez-Maciñeiras X., Bendicho-Lavilla C., Rial C., Garba-Mohammed K., Worsley A., Díaz-Torres E., Orive-Martínez C., Orive-Mayor A., Basit A.W., Alvarez-Lorenzo C. (2025). Advancing medication compounding: Use of a pharmaceutical 3D printer to auto-fill minoxidil capsules for dispensing to patients in a community pharmacy. Int. J. Pharm..

[4] Beer N., Hegger I., Kaae S., De Bruin M.L., Genina N., Alves T.L., Hoebert J., Kalvemark Sporrong S. (2021). Scenarios for 3D printing of personalized medicines—A case study. Explor. Res. Clin. Soc. Pharm..

[5] Chai S.Y.W., Phang F.J.F., Yeo L.S., Ngu L.H., How B.S. (2022). Future era of techno-economic analysis: Insights from review. Front. Sustain..

[6] Wang S., Chen X., Han X., Hong X., Li X., Zhang H., Li M., Wang Z., Zheng A. (2023). A Review of 3D Printing Technology in Pharmaceutics: Technology and Applications, Now and Future. Pharmaceutics.

[7] FabRx https://www.fabrx.co.uk/.

[8] Triastek https://www.triastek.com/.

[9] Aprecia https://www.aprecia.com/.

[10] DiHeSys https://www.digital-health-systems.com/.

[11] Dosermedical https://dosermedical.com/.

[12] van der Vossen A.C., Al-Hassany L., Buljac S., Brugma J.D., Vulto A.G., Hanff L.M. (2019). Manipulation of oral medication for children by parents and nurses occurs frequently and is often not supported by instructions. Acta Paediatr..

[13] Richey R.H., Hughes C., Craig J.V., Shah U.U., Ford J.L., Barker C.E., Peak M., Nunn A.J., Turner M.A. (2017). A systematic review of the use of dosage form manipulation to obtain required doses to inform use of manipulation in paediatric practice. Int. J. Pharm..

[14] Richey R.H., Shah U.U., Peak M., Craig J.V., Ford J.L., Barker C.E., Nunn A.J., Turner M.A. (2013). Manipulation of drugs to achieve the required dose is intrinsic to paediatric practice but is not supported by guidelines or evidence. BMC Pediatr..

[15] Zahn J., Hoerning A., Trollmann R., Rascher W., Neubert A. (2020). Manipulation of Medicinal Products for Oral Administration to Paediatric Patients at a German University Hospital: An Observational Study. Pharmaceutics.

[16] Batchelor H.K., Fotaki N., Klein S. (2014). Paediatric oral biopharmaceutics: Key considerations and current challenges. Adv. Drug Deliv. Rev..

[17] van den Anker J., Reed M.D., Allegaert K., Kearns G.L. (2018). Developmental Changes in Pharmacokinetics and Pharmacodynamics. J. Clin. Pharmacol..

[18] van Kampen E.E.M., Willemsteijn L., Ruijgrok E.J. (2022). 3D printing of drugs: Expanding the options for child-tailored pharmacotherapy. Arch. Dis. Child..

[19] Mangoni A.A., Jackson S.H. (2004). Age-related changes in pharmacokinetics and pharmacodynamics: Basic principles and practical applications. Br. J. Clin. Pharmacol..

[20] Hilmer S.N., McLachlan A.J., Le Couteur D.G. (2007). Clinical pharmacology in the geriatric patient. Fundam. Clin. Pharmacol..

[21] Darwich A.S., Polasek T.M., Aronson J.K., Ogungbenro K., Wright D.F.B., Achour B., Reny J.L., Daali Y., Eiermann B., Cook J. (2021). Model-Informed Precision Dosing: Background, Requirements, Validation, Implementation, and Forward Trajectory of Individualizing Drug Therapy. Annu. Rev. Pharmacol. Toxicol..

[22] Andreadis I.I., Gioumouxouzis C.I., Eleftheriadis G.K., Fatouros D.G. (2022). The Advent of a New Era in Digital Healthcare: A Role for 3D Printing Technologies in Drug Manufacturing?. Pharmaceutics.

[23] WHO Anatomical Therapeutic Chemical Classification. https://www.who.int/tools/atc-ddd-toolkit/atc-classification.

[24] Griens A.M.G.F., Kroon J.D.L., Lukaart J.S., Postma D.J., Verkroost M.J.S. (2020). SFK Data en Feiten 2020 Het Jaar 2019 in Cijfers.

[25] Bruyndonckx R., Adriaenssens N., Versporten A., Hens N., Monnet D.L., Molenberghs G., Goossens H., Weist K., Coenen S., the ESAC-Net study group (2021). Consumption of antibiotics in the community, European Union/European Economic Area, 1997–2017. J. Antimicrob. Chemother..

[26] Moor J.H. (2005). Why we need better ethics for emerging technologies. Ethics Inf. Technol..

[27] Hansson S.O. (2020). How extreme is the precautionary principle?. NanoEthics.

[28] KNMP Aantal Geneesmiddelentekorten over 2023 Hoger Dan Ooit. https://www.knmp.nl/actueel/nieuws/aantal-geneesmiddelentekorten-over-2023-hoger-dan-ooit.

[29] Rijksoverheid Leidraad Verantwoord Wisselen Medicijnen. https://open.overheid.nl/documenten/ronl-9783b4f97590d758f888567b76ad8aaaf029582d/pdf.

[30] KNMP KNMP-Handleiding Geneesmiddelensubstitutie. https://www.knmp.nl/sites/default/files/2022-02/Handleiding%20substitutie%202018.pdf.

[31] Richey R.H., Craig J.V., Shah U.U., Ford J.L., Barker C.E., Peak M., Nunn A.J., Turner M.A. (2012). The manipulation of drugs to obtain the required dose: Systematic review. J. Adv. Nurs..

[32] Ryu G.S., Lee Y.J. (2012). Analysis of liquid medication dose errors made by patients and caregivers using alternative measuring devices. J. Manag. Care Pharm..

[33] NKFK Kinderformularium. https://www.kinderformularium.nl/.

[34] Cipriani A., Furukawa T.A., Salanti G., Chaimani A., Atkinson L.Z., Ogawa Y., Leucht S., Ruhe H.G., Turner E.H., Higgins J.P.T. (2018). Comparative Efficacy and Acceptability of 21 Antidepressant Drugs for the Acute Treatment of Adults with Major Depressive Disorder: A Systematic Review and Network Meta-Analysis. Focus.

[35] Krause M., Gutsmiedl K., Bighelli I., Schneider-Thoma J., Chaimani A., Leucht S. (2019). Efficacy and tolerability of pharmacological and non-pharmacological interventions in older patients with major depressive disorder: A systematic review, pairwise and network meta-analysis. Eur. Neuropsychopharmacol..

[36] NHG NHG-Richtlijn Slaapproblemen. https://richtlijnen.nhg.org/standaarden/slaapproblemen.

[37] Bakker M.H., Hugtenburg J.G., van Straten A., van der Horst H.E., Slottje P. (2021). Effectiveness of low-dose amitriptyline and mirtazapine for insomnia disorder: Study protocol of a randomised, double-blind, placebo-controlled trial in general practice (the DREAMING study). BMJ Open.

[38] Harvey B.H., Slabbert F.N. (2014). New insights on the antidepressant discontinuation syndrome. Hum. Psychopharmacol..

[39] van Geffen E.C., Hugtenburg J.G., Heerdink E.R., van Hulten R.P., Egberts A.C. (2005). Discontinuation symptoms in users of selective serotonin reuptake inhibitors in clinical practice: Tapering versus abrupt discontinuation. Eur. J. Clin. Pharmacol..

[40] O’Brien C.P. (2005). Benzodiazepine use, abuse, and dependence. J. Clin. Psychiatry.

[41] Darker C.D., Sweeney B.P., Barry J.M., Farrell M.F., Donnelly-Swift E. (2015). Psychosocial interventions for benzodiazepine harmful use, abuse or dependence. Cochrane Database Syst. Rev..

[42] Van Leeuwen E., van Driel M.L., Horowitz M.A., Kendrick T., Donald M., De Sutter A.I., Robertson L., Christiaens T. (2021). Approaches for discontinuation versus continuation of long-term antidepressant use for depressive and anxiety disorders in adults. Cochrane Database Syst. Rev..

[43] Ibrahim K., Cox N.J., Stevenson J.M., Lim S., Fraser S.D.S., Roberts H.C. (2021). A systematic review of the evidence for deprescribing interventions among older people living with frailty. BMC Geriatr..

[44] Heneghan J.A., Trujillo Rivera E.A., Zeng-Treitler Q., Faruqe F., Morizono H., Bost J.E., Pollack M.M., Patel A.K. (2020). Medications for Children Receiving Intensive Care: A National Sample. Pediatr. Crit. Care Med..

[45] Balan S., Hassali M.A.A., Mak V.S.L. (2018). Two decades of off-label prescribing in children: A literature review. World J. Pediatr..

[46] Schrier L., Hadjipanayis A., Stiris T., Ross-Russell R.I., Valiulis A., Turner M.A., Zhao W., De Cock P., de Wildt S.N., Allegaert K. (2020). Off-label use of medicines in neonates, infants, children, and adolescents: A joint policy statement by the European Academy of Paediatrics and the European society for Developmental Perinatal and Pediatric Pharmacology. Eur. J. Pediatr..

[47] Glaeske G., Gerdau-Heitmann C., Hofel F., Schicktanz C. (2012). “Gender-specific drug prescription in Germany” results from prescriptions analyses. Sex and Gender Differences in Pharmacology.

[48] Orlando V., Mucherino S., Guarino I., Guerriero F., Trama U., Menditto E. (2020). Gender Differences in Medication Use: A Drug Utilization Study Based on Real World Data. Int. J. Environ. Res. Public Health.

[49] Fernandez-Liz E., Modamio P., Catalan A., Lastra C.F., Rodriguez T., Marino E.L. (2008). Identifying how age and gender influence prescription drug use in a primary health care environment in Catalonia, Spain. Br. J. Clin. Pharmacol..

[50] Roe C.M., McNamara A.M., Motheral B.R. (2002). Gender- and age-related prescription drug use patterns. Ann. Pharmacother..

[51] CBS Vrouwen Hebben Vaker Chronische Aandoeningen dan Mannen. https://www.cbs.nl/nl-nl/achtergrond/2011/10/vrouwen-hebben-vaker-chronische-aandoeningen-dan-mannen.

[52] CBS Meer Vrouwen dan Mannen met Psychische Klachten. https://www.cbs.nl/nl-nl/nieuws/2022/50/meer-vrouwen-dan-mannen-met-psychische-klachten.

[53] Ballering A.V., Muijres D., Uijen A.A., Rosmalen J.G.M., Olde Hartman T.C. (2021). Sex differences in the trajectories to diagnosis of patients presenting with common somatic symptoms in primary care: An observational cohort study. J. Psychosom. Res..

[54] SCP Vrouwen Leven Langer, Maar Zijn ze ook Gezonder?. https://digitaal.scp.nl/emancipatiemonitor2020/vrouwen-leven-langer-maar-zijn-ze-ook-gezonder/.

[55] Vogel B., Acevedo M., Appelman Y., Bairey Merz C.N., Chieffo A., Figtree G.A., Guerrero M., Kunadian V., Lam C.S.P., Maas A. (2021). The Lancet women and cardiovascular disease Commission: Reducing the global burden by 2030. Lancet.

